# Enhanced Brain Tumor Segmentation Using CBAM-Integrated Deep Learning and Area Quantification

**DOI:** 10.1155/ijbi/2149042

**Published:** 2025-08-01

**Authors:** Rafiqul Islam, Sazzad Hossain

**Affiliations:** Department of Computer Science and Engineering, Dhaka University of Engineering & Technology, Gazipur, Bangladesh

**Keywords:** area quantification, brain tumor analysis, lightweight U-Net model, magnetic resonance imaging, segmentation

## Abstract

Brain tumors are complex clinical lesions with diverse morphological characteristics, making accurate segmentation from MRI scans a challenging task. Manual segmentation by radiologists is time-consuming and susceptible to human error. Consequently, automated approaches are anticipated to accurately delineate tumor boundaries and quantify tumor burden, addressing these challenges efficiently. The presented work integrates a convolutional block attention module (CBAM) into a deep learning architecture to enhance the accuracy of MRI-based brain tumor segmentation. The deep learning network is built upon a VGG19-based U-Net model, augmented with depthwise and pointwise convolutions to improve feature extraction and processing efficiency during brain tumor segmentation. Furthermore, the proposed framework enhances segmentation precision while simultaneously incorporating tumor area measurement, making it a comprehensive tool for early-stage tumor analysis. Several qualitative assessments are used to assess the performance of the model in terms of tumor segmentation analysis. The qualitative metrics typically analyze the overlap between predicted tumor masks and ground truth annotations, providing information on the segmentation algorithms' accuracy and dependability. Following segmentation, a new approach is used to compute the extent of segmented tumor areas in MRI scans. This involves counting the number of pixels within the segmented tumor masks and multiplying by their area or volume. The computed tumor areas offer quantifiable data for future investigation and clinical interpretation. In general, the proposed methodology is projected to improve segmentation accuracy, efficiency, and clinical relevance compared to existing methods, resulting in better diagnosis, treatment planning, and monitoring of patients with brain tumors.

## 1. Introduction

Brain tumor is a major health concern around the world, with a wide range of morphological traits and clinical manifestations [1]. The accurate segmentation of brain tumors using magnetic resonance imaging (MRI) scans is critical for diagnosis, therapy planning, and patient monitoring. Traditional segmentation approaches frequently rely on radiologists' manual delineation, which can be time-consuming, subjective, and susceptible to interobserver variability [2]. Deep learning techniques, notably convolutional neural network (CNN), have recently transformed medical image processing by allowing for the automated and exact segmentation of anatomical features and pathological lesions from MRI scans [3]. These works have demonstrated the usefulness and efficiency of CNN, in automated brain tumor segmentation from MRI scans. Moreover, it offers useful insights, methodology, and benchmark results for researchers looking to build similar approaches for brain tumor investigation.

Over the last decade, various segmentation algorithms have been presented for the detection of anomalies such as tumors and gliomas from brain MRI utilizing image segmentation. In 2014, a Gaussian mixture model–based segmentation approach was proposed by the authors [4] for brain MRI images. Recently, multiple studies have demonstrated the usefulness and efficiency of deep learning, particularly CNN-based approaches, for automated brain tumor segmentation from MRI scans [5]. These studies offer valuable insights, methodology, and benchmark results for researchers looking to build similar approaches for brain tumor investigation. Researchers have explored various modifications to CNN models, such as incorporating symmetric masks [6], leveraging ensemble architectures combining CNN and U-Net for BraTS2019 [7], and integrating SVM with CNN for BraTS-2018 [8]. Furthermore, a cascade deep learning model was proposed to determine tumor stages, assisting medical professionals [9]. A hybrid U-Net-based feature extraction model was introduced to find the complex anatomical structures and low discriminative features and applied on the BraTS and LGG-MRI datasets for the segmentation task [10]. Several performance metrics and experimental analyses were performed to validate the model. In 2023, a hybrid feature extraction model was designed by incorporating residual U-Net, attention, and squeeze–excitation modules to perform the segmentation [11], and for experimental analysis, publicly available BraTS-2020 dataset was employed. An enhanced U-Net architecture was presented in [12] that considered the inception block rather than the convolutional block during the skip connection phase utilizing multiple BraTS datasets for segmentation. Edge U-Net, constructed with an encoder-decoder framework from the U-Net architecture, was launched and customized for brain tumor segmentation using a T1-weighted MRI image dataset [13]. It integrates boundary-related information with basic MRI images to increase tumor localization accuracy. Segmentation and survival rate prediction were investigated in the same year, with a multimodal MRI BraTS dataset employed to examine the tumor for diagnosis and treatment planning [14]. In their work, they developed a hybrid deep learning architecture incorporating a convolutional normalized mean filter for preprocessing and a residual recurrent U-Net segmentation model. In [15], the authors developed a technique for brain tumor segmentation and survival prediction based on radiomics properties collected from segmented tumor regions. The work illustrates the effectiveness of deep learning–based segmentation approaches in conjunction with radiomics analysis for predicting patient outcomes and risk stratification in glioma patients. Havaei et al. [16] suggested a rapid and accurate technique for brain tumor segmentation that uses both local and broader contextual characteristics from the BraTS-2013 dataset. Naser and Deen [17] designed a segmentation framework based on U-Net and a pretrained VGG16 model to simultaneously segment, detect, and grade LGG tumors using the BraTS dataset. The Alzheimer's Disease Neuroimaging Initiative (ADNI) [18] and Open Access Series of Imaging Studies (OASIS) are other popular MRI datasets used for classification and segmentation specially for dementia cases [19–21]. The authors of [19] used the dataset and introduced multiple ensemble models in their study for characterizing and estimating Alzheimer's disease, a progressive neurological disorder. Ensemble deep learning approaches have shown promise in improving the accuracy and reliability of Alzheimer's disease diagnosis by integrating multiple data modalities and leveraging advanced machine learning techniques. In [20], the authors analyzed different models for this dataset that merged fuzzy logic with several deep learning and explainable artificial intelligence models. The objective behind these models was to handle more accurate and linguistically interpretable decision support systems for detection, segmentation, and classification.

According to the research, U-Net-based deep learning frameworks and the BraTS dataset are the most effective and commonly used for segmentation. Despite substantial advances in deep learning–based image segmentation, precisely defining brain tumors remains challenging, particularly in the face of diverse tumor morphology, fluctuating image contrast, and noise distortions. Furthermore, while segmentation gives useful information about tumor location and extent, quantifying the tumor burden using measures like tumor area is critical for determining disease progression, treatment response, and prognosis. This work presents accurate and efficient deep learning models for brain tumor segmentation from MRI scans, as well as methods for calculating tumor area. These methodologies are aimed at improving segmentation accuracy, efficiency, and clinical relevance compared to existing methods, resulting in better diagnosis, treatment planning, and monitoring of patients with brain tumors. In summary, possible contributions of the works are as follows:

• To design a convolutional block attention module (CBAM) with a VGG19-based U-Net architecture that has been augmented with depthwise and pointwise convolutions to boost feature extraction and processing efficiency during brain tumor segmentation.
• To develop an algorithm for calculating the area of segmented tumor regions in MRI scans, providing quantitative measurements for further analysis.
• Exploration of the clinical relevance of segmented tumor areas, highlighting their potential for aiding in diagnosis, treatment planning, and outcome prediction.

## 2. Theory and Method

MRI is a popular medical imaging technology that generates comprehensive images of soft tissues within the human body. Accurate segmentation of MRI images, especially for brain tumors, is crucial for diagnosis and treatment. Traditional segmentation approaches, such as thresholding, are limited in their capacity to handle the complexity and variability of MRI data. In contrast, deep learning techniques have demonstrated exceptional performance in medical image segmentation [22]. Among them, the U-Net architecture and its variations have grown in prominence due to their capacity to provide reliable segmentations despite low training data.

### 2.1. Related Works

Segmentation is the strategy of dividing an image into individual regions or entities. After effective image segmentation, objects can be identified based on their shape, texture, and color. The segmentation algorithm discovers abrupt changes in intensity, such as edges, and partitions regions based on predefined criteria.

#### 2.1.1. Traditional Methods

Thresholding-based segmentation is most commonly used in classical image processing techniques. It relies on pixel intensity comparisons to differentiate foreground and background regions. These approaches are employed for image segmentation, with each pixel value being assessed according to the rule:

(1) $$\begin{array}{c} g\left(x,y\right)=\left\{\begin{array}{cc} 0, & f\left(x,y\right)<T\\ 1, & f\left(x,y\right)\geq T, \end{array}\right. \end{array}$$

where *T* is the threshold value. This equation is widely incorporated into different segmentation algorithms. However, finding the optimal threshold value (*T*) is the most challenging task.

• **Iterative thresholding:** The iterative self-organizing data analysis technique algorithm (isodata) is one of the most prominent ways to find the optimal threshold value [23] in which the working structure of the model can be described as follows:
  1. Compute *μ*_1_ and *μ*_2_ from the foreground and background pixels, respectively.
  2. *T*_new_ = (*μ*_1_ + *μ*_2_)/2.
  3. Loop (until *T*_new_ ≠ *T*_old_) do
  4. Compute *μ*_1_ and *μ*_2_ from the foreground and background pixels, respectively, using new  *T*_new_.
  5. *T*_old_ = *T*_new_,
  6. *T*_new_ = (*μ*_1_ + *μ*_2_)/2.
  7. End Loop

*T*_new_ is the final optimal threshold value.

• **Ostu thresholding:** The Otsu approach uses clustering to determine image thresholds, and it works with bimodal histograms [24] on the MRI for tumor segmentation. The approach essentially attempts to decrease within-class variance while maximizing between-class variance.

 $$\begin{array}{c} \text{Total variance}=\text{within}-\text{class variance}+\text{between}-\text{class variance}. \end{array}$$

Overall, the model can be written as follows:

1. Create histogram from the gray scale image
2. Based on threshold value (*T*), separated the image into two classes: Class 1 (value < = *T*) and Class 2 (value > *T*)
3. Compute variance: *σ*^2^ = ∑(*X*_*i*_ − *μ*)^2^/*N*
4. Compute within class variance

#### 2.1.2. Deep Learning–Based Approaches

U-Net is a standard deep learning model that was introduced in 2015, and it was introduced for biomedical image segmentation. Till now, it is the most widely used model due to its ability to achieve precise pixel-wise segmentation with limited annotated data [25]. The characteristic “U” shape of the architecture is created by the symmetric expanding and contracting channels. The fundamental purpose of this architecture is to solve the medical industry's lack of annotated data, and it was designed to consume less data while remaining fast and accurate.

**Figure 1:**
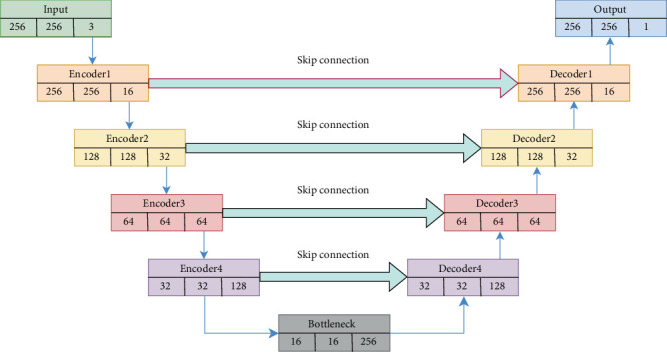
Block diagram of U-Net model.

The U-Net architecture comprises two channels: the encoder, which contracts, and the decoder, which expands. The encoder mixes convolutional and pooling layers to capture context while reducing spatial dimensions. The decoder expands the feature maps before combining them with the encoder's associated high-resolution features via skip connections. This structure enables the network to learn both global and local properties efficiently. The overall architecture is presented in Figure 1.

Several improvements of U-Net model has been done by integrating residual blocks from ResNet [26], lightweight features from EfficientNet [27], densely connected layers inspired by DenseNet [28]. Each of these U-Net versions expands on the basic architecture, addressing various challenges: the residual network solves the vanishing gradient problem and allows for the formation of deeper networks capable of learning complex characteristics, multiscale feature aggregation of EfficientNet layers improves the algorithm's efficiency and suitability for resource-constrained applications, and Dense U-Net effectively reuses features and creates a consistent gradient flow, reducing the likelihood of gradients disappearing. These developments, taken together, help to improve the reliability and precision of medical picture segmentation systems.

#### 2.1.3. Hybrid Network–Based Approaches

**Figure 2:**
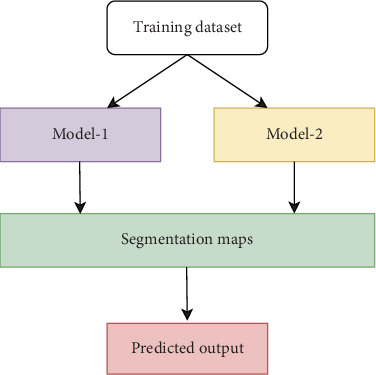
Block diagram of hybrid networks.

Ensembling segmentation using CNN and U-Net is a powerful approach in medical imaging, particularly for tasks like brain tumor segmentation. This technique utilizes the capabilities of both designs to improve segmentation accuracy and reliability. Generally, CNN is used to extract features for identifying patterns, whereas the U-Net allows capturing spatial and contextual information to make precise pixel-level segmentation. Each model produces segmentation maps, which are then combined to yield the final prediction. This combination can be achieved using techniques such as weighted averaging or choosing the best-performing model for a specific subregion. The block diagram of the ensemble model for segmentation is presented in Figure 2.

## 3. Proposed Segmentation and Area Quantification Method

**Figure 3:**
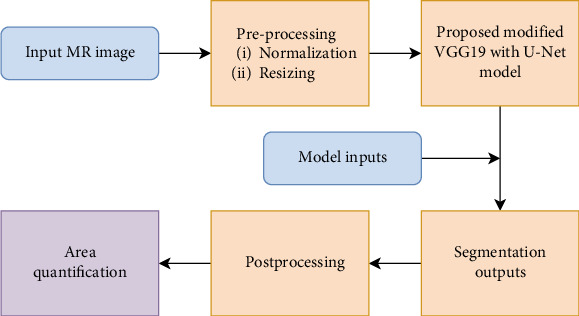
Basic block diagram of the proposed model.

The suggested segmentation algorithms, which are based on a modified VGG19-based U-Net architecture, constitute a powerful approach to MRI segmentation by combining the structural advantages of U-Net with the optimization benefits of attention learning. Its capacity to collect complex information, manage deep networks, and give accurate segmentation makes it an important tool in medical imaging, especially for difficult tasks like brain tumor segmentation. This architecture not only improves segmentation accuracy but also increases the clinical value of MRI for diagnosing and treating brain cancers. The architecture of the proposed algorithm is presented in Figure 3.

### 3.1. Preprocessing

Preprocessing utilizes intensity normalization, skull stripping, and image registration as critical for increasing the quality and consistency of MRI images prior to segmentation. To reduce the computational complexity of the model, the images are scaled to the range of [0, 1] using the formula below:

(2) $$\begin{array}{c} I_{N}=\frac{I\left(x,y\right)-\text{Min}\left(I\right)}{\text{Max}\left(I\right)-\text{Min}\left(I\right)}, \end{array}$$

where *I*(*x*, *y*) represents the pixel value of the image. Data augmentation processes like rotation, scaling, and mirroring can strengthen deep learning models and increase their generalization performance. For augmentation, rotation and flipping are explored, with 45°, 90°, and 135° used to enhance the number of images in the training dataset.

### 3.2. Proposed Modified VGG19 With U-Net Model

**Figure 4:**
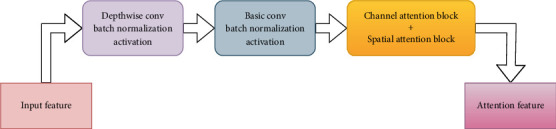
Proposed CBAM architecture.

This work proposes a deep learning architecture leveraging the VGG19-based U-Net model to efficiently and accurately segment brain tumors from MRI images. The model seeks to reduce computational complexity, improve segmentation accuracy, and shorten inference time while retaining a compact design with fewer parameters. The model keeps the fundamental ideas of the U-Net design, including an encoder–decoder structure with skip links, but makes many changes to improve efficiency. The encoder is made up of several convolutional blocks from the pretrained VGG19 network [29] that gradually downsample the input image while extracting high-level features. To prevent overfitting, a dropout layer is added after each convolutional block. The decoder mirrors the encoder but conducts upsampling rather than downsampling utilizing the CBAM [30]. The decoder model includes a CBAM employing depthwise and pointwise separable convolution layers, which improve feature extraction and processing efficiency during brain tumor segmentation. The architectural block diagram of the CBAM network is illustrated in Figure 4. Each upsampling procedure incorporates transposed convolutions, which improve the spatial resolution of the feature maps. Skip connections from the respective encoder layers are combined with the upsampled feature maps to preserve spatial information and ensure accurate localization.

The bottleneck layer works as a bridge between the encoder and decoder, holding the most significant filters and doing substantial feature extraction. The final layer is a 1 × 1 convolution, which lowers the number of channels to one and creates a binary segmentation mask that is the same size as the input image. A Sigmoid activation function is used to calculate pixel-wise probabilities for the existence of the tumor.

**Figure 5:**
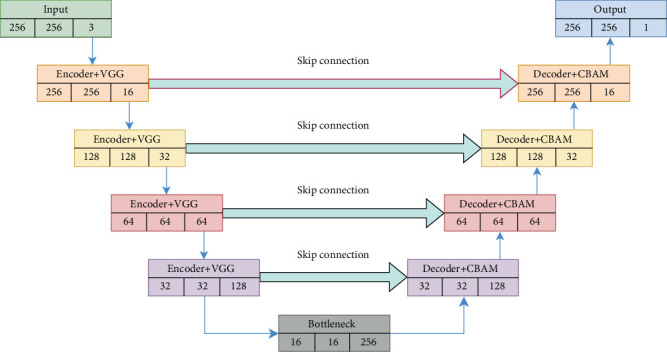
Proposed modified VGG19-based U-Net with CBAM network.

In this paper, CBAM processes are integrated into a U-Net architecture while keeping the model lightweight. The main advantage is that it can help focus on the most important spatial features during reconstruction, improving segmentation accuracy. These blocks are often integrated between the encoder's skip connections and the appropriate upsampled features in the decoder. The model's key advantage is the use of depthwise separable convolutions, which substantially reduce the amount of parameters and computational costs when compared to traditional convolutions, resulting in a lighter and faster model. Furthermore, skip connections assure that spatial resolution is maintained during upsampling, resulting in exact segmentation of tumor boundaries. The proposed modified VGG19-based U-Net model with CBAM architecture of the algorithm is presented as below Figure 5:

### 3.3. Area Quantification

**Figure 6:**
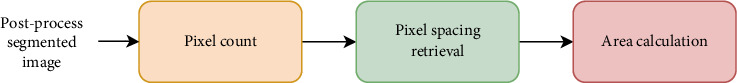
Proposed area quantification flow diagram.

This study presents a segmentation model followed by tumor area calculation, which is essential for assessing tumor growth, treatment efficacy, and prognosis. Tumor area measurement plays a significant role in clinical decision-making, as it is closely associated with disease severity and patient outcomes. By integrating these measurements with clinical data, specialists can analyze their relevance for surgical and therapeutic planning. Additionally, tracking segmented tumor areas from multiple MRI scans allows medical professionals to monitor disease progression over time. Furthermore, tumor area quantification enables exploration of correlations with key prognostic indicators, such as tumor grade, patient survival, and therapy response. It is the process of converting a segmented region's pixel count into a physical area measurement based on pixel spacing information from MRI metadata. The flow diagram of area quantification is shown in Figure 6.

#### 3.3.1. Pixel Counting

The number of pixels of a tumor from the segmented image can be calculated using the following formula:

(3) $$\begin{array}{c} N_{P}=\overset{M}{\underset{i=0}{\sum }}\overset{N}{\underset{j=0}{\sum }}I\left(i,j\right), \end{array}$$

where *I* indicate the segmented image with the desirable tumor sizes *M* and *N*. Here, *I*(*i*, *j*) = 1 denotes the pixel corresponding to the tumor region, while *I*(*i*, *j*) = 0 represents the remaining pixel values.

#### 3.3.2. Physical Area Calculation

MRI metadata that includes pixel spacing values, which represent the physical dimensions of each pixel in the MRI image. Let *S*_*x*_ and *S*_*y*_ be the pixel spacing values in the *x* and *y* directions, respectively. The pixel's physical area is given by the following:

(4) $$\begin{array}{c} P_{A}=S_{x}\times S_{y}. \end{array}$$

The physical area of the segmented tumor in square millimeters (mm^2^) is calculated by combining the pixel's physical area (*P*_*A*_) with the total number of pixels (*N*_*P*_).

(5) $$\begin{array}{c} \text{Area}=P_{A}\times N_{P}. \end{array}$$

## 4. Experimental Results

The suggested algorithm was validated for tumor segmentation and area quantification using brain MRI data. The key goals of this analysis were to evaluate the model's accuracy, efficiency, and segmentation quality, as well as to compare it to traditional U-Net designs.

### 4.1. Dataset

**Figure 7:**
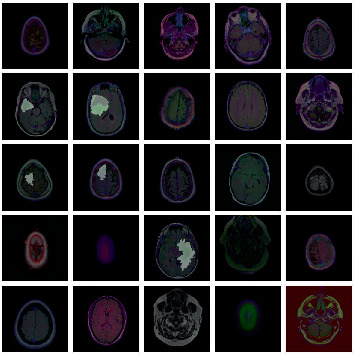
Few sample images.

To conduct the investigation, publicly accessible BraTS datasets (Version 3) from the multimodal brain tumor segmentation challenge were used as a benchmark dataset for evaluating tumor segmentation algorithms [31–33]. The dataset represents 110 lower grade glioma (LGG) patients from the Cancer Genome Atlas collection that have sufficient fluid-attenuated inversion recovery (FLAIR) sequence and genomic cluster data available. It includes brain MR images and customized FLAIR abnormality segmentation masks taken from the Cancer Imaging Repository. These datasets contain MRI images of various tumor types, sizes, and locations, allowing for a full study of segmentation algorithms under different conditions.  Figure 7 exhibits a few tumor and nontumor images from the dataset.

The BraTS collection, which includes multiparametric MRI images such as contrast-enhanced T1, T2, and FLAIR sequences, is primarily intended for brain tumor investigation. In contrast, datasets such as ADNI and OASIS focus on the analysis of neurodegenerative disorders like Alzheimer's [34]. Since this work primarily focuses on tumor segmentation and area quantification, the BRaTS dataset is considered here for the experimental evaluation and analysis.

### 4.2. Complexity Analysis

This work makes use of the Keras flops and parameter counts libraries for model complexity analysis.  Table 1 displays the total number of trainable parameters (TPs), nontrainable parameters (NTPs), and floating point operations per second (FLOPs) for all models utilized here.

The investigation revealed that VGG19 and Residual U-Net have TP values of approximately 31 M and 41 M, respectively, which are higher than those of any other models. On the other hand, EfficientNet and DenseNet with U-Net have around 24 M parameters, making them more compact than the preceding two models. In contrast, the proposed model has the lowest TP (20.4 M), making it the lightest among all variants. The NTP values indicate that all other models utilize pretrained feature extractors with frozen layers. Although the proposed model is parameter-efficient, its computational cost in terms of FLOPs remains moderate. While it is not the lightest model overall, it achieves a balance between computational complexity and efficiency.

### 4.3. Preprocessing

The database contains 7858 brain MRI images, each with three RGB channels. Each image was scaled to 256 × 256 pixels using nearest neighbor interpolation and normalized to [0, 1]. The dataset is divided into three components: 70% for training, 15% for validation, and the remaining part for testing. Data augmentation was applied to increase the diversity of training images, incorporating random rotations (0.2), shifts (0.05), and zooms (0.05) with the nearest neighbor fill mode, which helps reduce overfitting. The model was trained for 150 epochs using the Adamax optimizer, a batch size of 40, and an initial learning rate of 0.00025. Additionally, a callback system was implemented to improve the model based on dice loss, ensuring better performance and stability during training. An early stopping mechanism with a tolerance of 15 epochs was adopted, and the best model was selected according to validation accuracy. To account for GPU memory limits, the model was trained employing variable precision.

### 4.4. Evaluation Metrics

The performance of segmentation algorithms is statistically assessed using measures such as accuracy, Dice coefficients (DSCs), and intersection over union (IoU).

1. Accuracy: Accuracy is the proportion of pixels properly categorized across all classes in an image. This metric measures how effectively it divides an image into discrete segments.

 $$\begin{array}{c} \text{Accuracy}=\frac{\text{Number of Correct Pixels}}{\text{Total Number of Pixels}}. \end{array}$$

2. DSCs: DSC reveal the similarity of predicted segmentation with traditionally segmented output labels. It is similar to the *F*1 score and considers equally true and false positives, resulting in its more accommodating in specific circumstances.

 $$\begin{array}{c} \text{DSC}=\frac{2\times \left|P\cap O\right|}{\left|P\right|+\left|O\right|}. \end{array}$$

3. IoU: IoU indicates the overlap between the predicted segmentation mask and the ground truth mask. The IoU value is computed by dividing the expected and actual masks' intersection area by their union area. This metric is used to evaluate the accuracy and efficacy of segmentation models in detecting and distinguishing objects within images.

 $$\begin{array}{c} \text{IoU}=\frac{\left|P\cap O\right|}{\left|P\cup O\right|}, \end{array}$$

where *P* and *O* are the estimated and manually segmented output label, respectively.

### 4.5. Segmentation Performance

**Figure 8:**
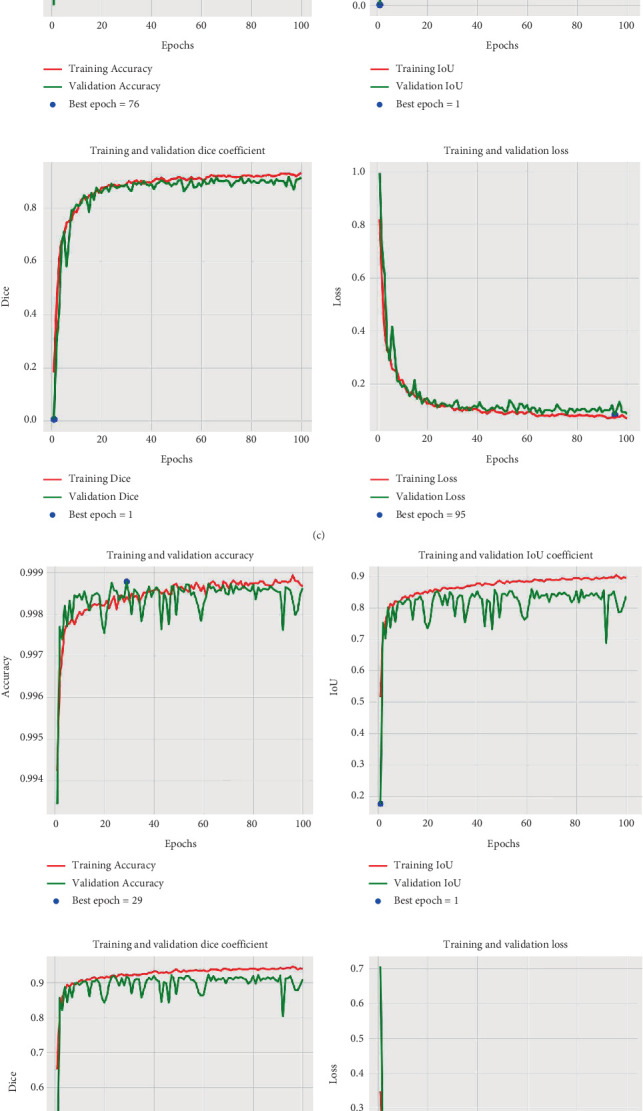
IoU, Dice coefficient, and loss curve of (a) U-Net-based, (b) ResNet with U-Net–based, (c) VGG19 with U-Net–based, (d) EfficientNet with U-Net model, (e) DenseNet with U-Net model, and (f) proposed VGG19 with U-Net–based.

Significant accuracy and efficiency are key in the field of segmentation, which also play important roles in algorithm evaluation. Although transformer-based segmentation models and self-supervised learning methods have demonstrated strong performance, this study primarily focuses on evaluating the effectiveness of U-Net-based approaches on the BRaTS dataset. Therefore, U-Net-based approaches were selected for comparison and evaluation. This paper presents a comprehensive analysis of several cutting-edge artificial neural network models, including U-Net, ResNet–U-Net, VGG19–U-Net, EfficientNet–U-Net, DenseNet–U-Net, and the proposed model. The performance of these models was assessed across training, validation, and testing datasets utilizing important metrics such as loss, accuracy, IoU, and DSC.  Figure 8 depicts the training and validation curves, which provide critical insights into the model's learning process, ensuring that the model is both correct and generalizable.

The figure demonstrates that the suggested algorithm surpasses all standard models in terms of DSC, IoU, and loss. It achieves the highest segmentation accuracy with an IoU of 0.8940 and the lowest loss of 0.0561. The DenseNet with U-Net model algorithm follows closely with an IoU of 0.8864 and a loss of 0.0606. The EfficientNet with U-Net model also performs well, achieving an IoU of 0.8766 and a loss of 0.0661. The VGG19 with U-Net model shows significant improvement over the basic U-Net and Residual U-Net models, with an IoU of 0.8799 and a loss of 0.0643. The residual U-Net model has an IoU of 0.8147 and a loss of 0.1037, while the basic U-Net model has an IoU of 0.8204 and a loss of 0.0999. Furthermore, both the training and validation curves drop and plateau at identical places, indicating that the model is learning efficiently and without overfitting.

To effectively illustrate the superior outcome of the suggested brain tumor segmentation model, we organized the results for the training, validation, and testing datasets of different state-of-the-art approaches that are provided in Tables 2, 3, and 4.

Tables shows that the proposed algorithm consistently achieves the lowest loss values (0.0561 in training, 0.0855 in validation, and 0.1066 in testing), indicating it makes fewer errors compared to other models. It also shows the highest accuracy (0.9989 in training, 0.9983 in validation, and 0.9984 in testing), demonstrating its superior ability to make correct predictions. Additionally, the proposed model achieves the highest IoU (0.8940 in training, 0.8434 in validation, and 0.8088 in testing), showing it has the best overlap with the actual data. Finally, it achieves the highest DSC (0.9437 in training, 0.9146 in validation, and 0.8935 in testing), indicating it has the highest similarity to the ground truth. These metrics collectively highlight the proposed model's efficiency in learning and its robustness in segmentation tasks, demonstrating noticeable improvement over state-of-the-art methods. As demonstrated by the results, the improvement in training accuracy is marginal; however, the improvement in test results is significantly noticeable, exceeding 2%.

In a nutshell, the suggested modified VGG19- and CBAM-based U-Net model was effective in segmenting brain tumors from MRI images. The DSC and IoU scores were both very high, indicating accurate and consistent segmentation results. These results highlight the suggested model's stability and efficiency, making it a feasible option for advanced segmentation tasks.

### 4.6. Statistical Analysis

To comprehensively analyze the suggested strategy, this study uses statistical analysis, including standard deviation (SD), to provide more information about the model's performance and reliability. The statistical analysis (mean ± SD) is limited to the suggested model, as it is the primary contribution of this study, and assessing its consistency provides a better understanding of its reliability and effectiveness. The accuracy, IoU, and DSC of the proposed model were evaluated over seven trials in the statistical analysis conducted on training, validation, and test cases. The average scores are presented in Table 5. However, test cases are the primary focus of the analysis, as they provide the most reliable indication of the algorithm's performance. According to the results, the average accuracy, IoU, and DSC for the test cases are 0.9982 ± 0.000124, 0.8270 ± 0.0082, and 0.9046 ± 0.0050, respectively. These results suggest that the model's segmentation performance is both reliable and consistent.

### 4.7. Segmentation Results

**Figure 9:**
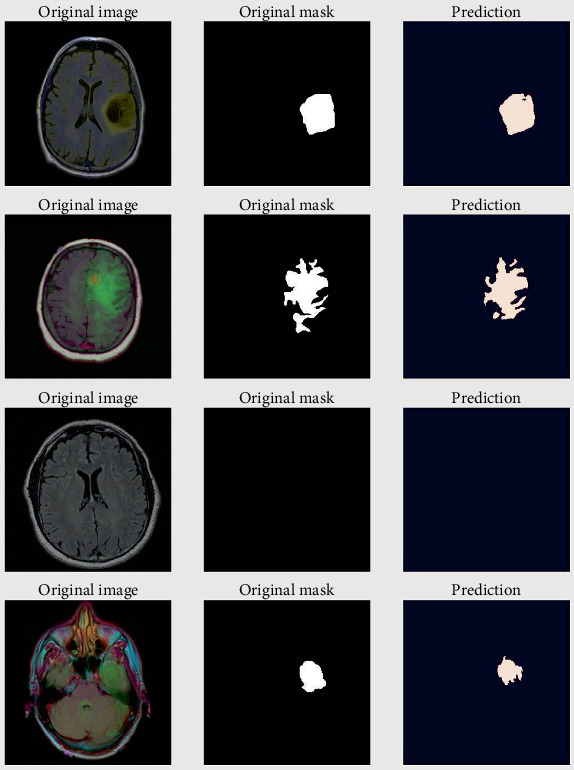
Segmentation result of the proposed algorithm.

Figure 9 depicts the visual representation of the suggested method. The figure shows that the suggested model accurately segmented the tumor from the MRI images. However, some tumors cannot be recognized using the suggested approach because the features of benign tumors are not well highlighted in MRI images.

**Figure 10:**
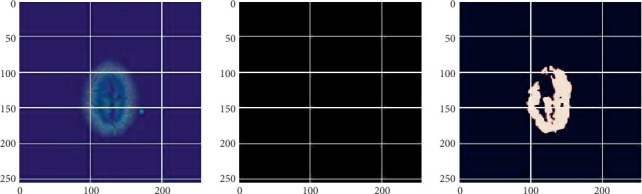
Failure case from the segmentation results of the proposed algorithm.

Although the quantitative and qualitative study of the segmentation results reveals the model's success in certain circumstances, there are some specific characteristics of tumors where the model exhibits difficulty in segmenting as shown in Figure 10. Figure 10 demonstrates that the model confronts issues for intensity volatility, low contrast, and poorly defined boundaries of a tumor. Furthermore, the model has difficulties when the image contains much more background pixels than foreground pixels.

### 4.8. Area Quantification

Accurate measurement of the tumor area is critical for clinical analysis and therapy planning. There is frequently an association between tumor size and cancer grade. Higher grade cancers grow faster and may cover larger areas. Accurate tumor area measurement can aid in grading the malignancy, which is critical for prognosis and therapy planning. The tumor area might also indicate the severity of the disease. Larger tumor regions are typically associated with a worse prognosis, as they may signify more advanced disease.

**Figure 11:**
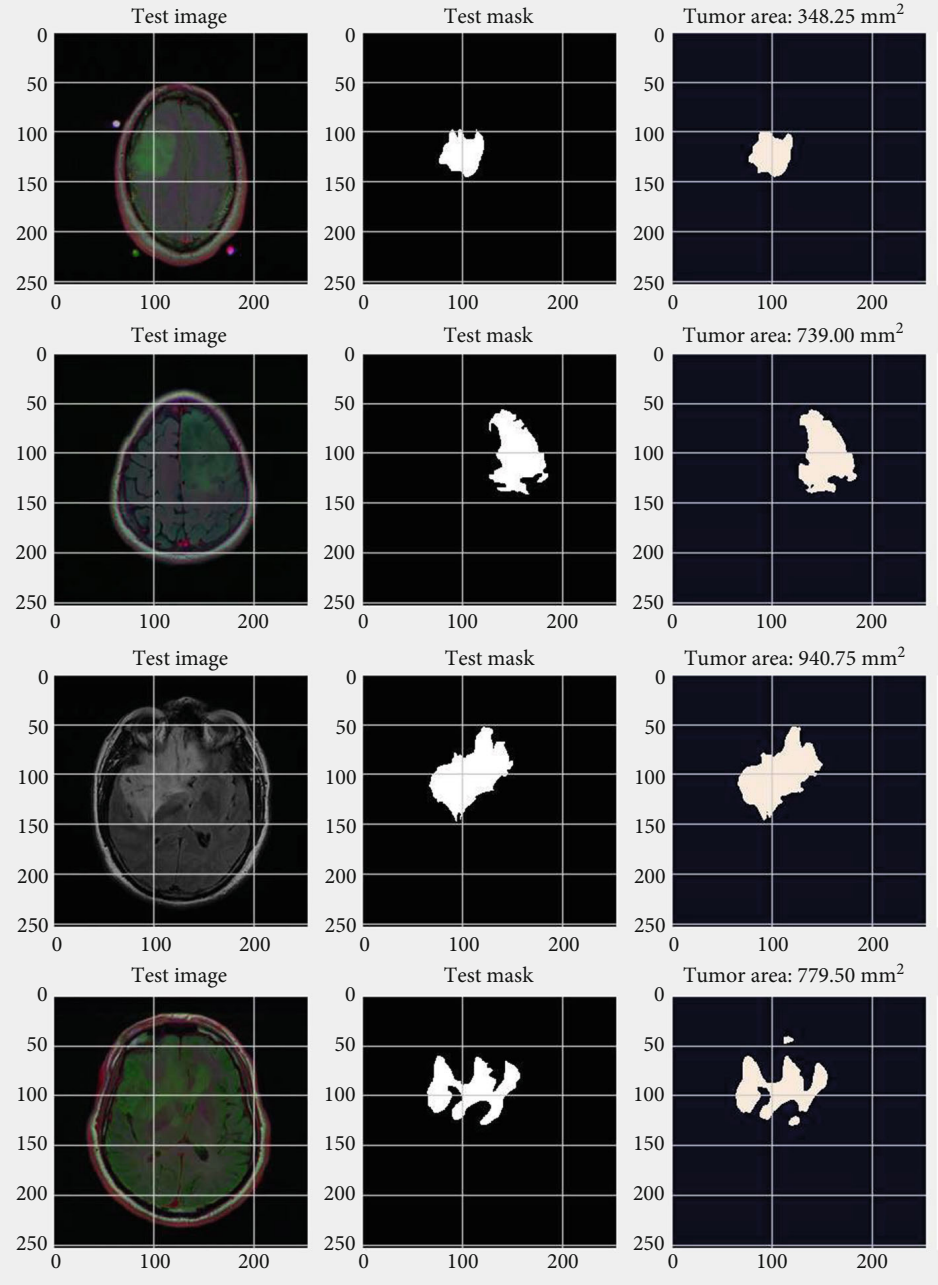
Area quantification from the segmented tumor in squared millimeters in a CBAM, spanning training, validation, and testing datasets. These findings demonstrate the model's noticeable capacity to effectively segment tumors, reduce mistakes, and remain very similar to ground truth annotations. The addition of tumor area assessment increases its clinical utility, giving precise and measurable data for diagnosis, treatment planning, and patient monitoring. Overall, the suggested model's efficiency, precision, and resilience make it an important tool for improving early-stage brain tumor diagnosis and therapeutic outcomes. Despite the positive results obtained by the provided model, its current capabilities are limited for certain clinical applications, such as reliably detecting cancer grade. Additional modifications are required to improve its effectiveness and dependability in such crucial medical situations.

To find the tumor area, the segmented regions were transformed into tumor sizes using the pixel spacing information from the MRI scans. Each MRI slice was assigned a binary mask that indicated the existence of tumor regions, and the number of pixels corresponding to the tumor region in each mask was counted. The pixel count has been mapped to actual area using the pixel spacing values from the MRI images' DICOM metadata. The model consistently produces therapeutically meaningful values, as seen in Figure 11, which depicts the tumor area from a few segmented tumors.

## 5. Conclusion

This work offered a modified VGG19-based U-Net algorithm for brain tumor segmentation, followed by area quantification. Several investigations were accomplished to validate the proposed model, and the results were compared to standard state-of-the-art models regarding segmentation performance. The model obtained improved performance metrics by combining depthwise and pointwise separable convolutions.

## Figures and Tables

**Table 1:** Total parameters (trainable and non-trainable) and FLOPs of all the models.

**Evaluation metrics**	**U-Net model**	**Residual U-Net model**	**VGG19 with U-Net**	**EfficientNet with U-Net**	**DenseNet with U-Net**	**Proposed model**
TP	31.0 × 10^6^	41.0 × 10^6^	31.2 × 10^6^	24.0 × 10^6^	24.9 × 10^6^	20.4 × 10^6^
NTP	5888	56,960	3840	153,872	225,216	2880
FLOPs	3.9 × 10^12^	1.3 × 10^12^	4.5 × 10^12^	0.5 × 10^12^	0.8 × 10^12^	2.1 × 10^12^

**Table 2:** Comparison of the proposed method with state-of-the-art methods (training).

	**U-Net model**	**ResNet with U-Net**	**VGG19 with U-Net**	**EfficientNet with U-Net**	**DenseNet with U-Net**	**Proposed model**
Loss	0.0999	0.1037	0.0643	0.0661	0.0606	0.0561
Accuracy	0.9981	0.9981	0.9987	0.9987	0.9987	0.9989
IoU	0.8204	0.8147	0.8799	0.8766	0.8864	0.8940
DSC	0.9001	0.8965	0.9358	0.9339	0.9395	0.9437

**Table 3:** Comparison of the proposed method with state-of-the-art methods (validation).

	**U-Net model**	**ResNet with U-Net**	**VGG19 with U-Net**	**EfficientNet with U-Net**	**DenseNet with U-Net**	**Proposed model**
Loss	0.1265	0.1252	0.0928	0.1005	0.0975	0.0855
Accuracy	0.9975	0.9975	0.9985	0.9985	0.9981	0.9983
IoU	0.7780	0.7801	0.8320	0.8196	0.8236	0.8434
DSC	0.8735	0.8752	0.9073	0.8996	0.9023	0.9146

**Table 4:** Comparison of the proposed method with state-of-the-art methods (test).

	**U-Net model**	**ResNet with U-Net**	**VGG19 with U-Net**	**EfficientNet with U-Net**	**DenseNet with U-Net**	**Proposed model**
Loss	0.1089	0.1560	0.0846	0.1161	0.0964	0.1066
Accuracy	0.9976	0.9973	0.9982	0.9978	0.9981	0.9984
IoU	0.8050	0.7345	0.8450	0.7935	0.8256	0.8088
DSC	0.8907	0.8440	0.9155	0.8837	0.9038	0.8935

**Table 5:** Statistical analysis (mean ± SD) of the proposed algorithm.

	**Accuracy**	**IoU**	**Dice**
Training	0.9987 ± 0.000248	0.8785 ± 0.01952	0.9349 ± 0.0110
Validation	0.9984 ± 0.000026	0.8284 ± 0.0103	0.9054 ± 0.0063
Test	0.9982 ± 0.000124	0.8270 ± 0.0082	0.9046 ± 0.0050

## Data Availability

Data sharing is not applicable to this article as no new data were created or analyzed in this study.
